# Abnormalities in fronto-striatal connectivity within language networks relate to differences in grey-matter heterogeneity in Asperger syndrome^☆^

**DOI:** 10.1016/j.nicl.2013.05.010

**Published:** 2013-05-27

**Authors:** Eugenia Radulescu, Ludovico Minati, Balaji Ganeshan, Neil A. Harrison, Marcus A. Gray, Felix D.C.C. Beacher, Chris Chatwin, Rupert C.D. Young, Hugo D. Critchley

**Affiliations:** a Psychiatry, Brighton & Sussex Medical School (BSMS), Brighton, BN1 9RY, UK; b Sackler Centre for Consciousness Science, University of Sussex, Brighton, BN1 9RY, UK; c Neurology and Psychiatry, Brighton & Sussex Medical School (BSMS), Brighton, BN1 9RY, UK; d U.O. Direzione Scientifica, Fondazione IRCCS Istituto Neurologico Carlo Besta, Milano, Italy; e University College London (UCL), Institute of Nuclear Medicine, London, NW1 2BU, UK; f Sussex Partnership NHS Foundation Trust, c/o Sussex Education Centre, Mill View Hospital Site, Hove, BN3 7HZ, UK; g University of Queensland, Centre for Advanced Imaging, Gehrmann Laboratory, Brisbane, 4072, Australia; h Department of Engineering and Design, University of Sussex, BN1 9QT, UK

**Keywords:** Autism Spectrum Disorder (ASD), Texture analysis (TA), Psycho-physiological interaction (PPI), Dynamic causal modelling (DCM), Caudate

## Abstract

Asperger syndrome (AS) is an Autism Spectrum Disorder (ASD) characterised by qualitative impairment in the development of emotional and social skills with relative preservation of general intellectual abilities, including verbal language. People with AS may nevertheless show atypical language, including rate and frequency of speech production. We previously observed that abnormalities in grey matter homogeneity (measured with texture analysis of structural MR images) in AS individuals when compared with controls are also correlated with the volume of caudate nucleus. Here, we tested a prediction that these distributed abnormalities in grey matter compromise the functional integrity of brain networks supporting verbal communication skills. We therefore measured the functional connectivity between caudate nucleus and cortex during a functional neuroimaging study of language generation (verbal fluency), applying psycho-physiological interaction (PPI) methods to test specifically for differences attributable to grey matter heterogeneity in AS participants. Furthermore, we used dynamic causal modelling (DCM) to characterise the causal directionality of these differences in interregional connectivity during word production. Our results revealed a diagnosis-dependent influence of grey matter heterogeneity on the functional connectivity of the caudate nuclei with right insula/inferior frontal gyrus and anterior cingulate, respectively with the left superior frontal gyrus and right precuneus. Moreover, causal modelling of interactions between inferior frontal gyri, caudate and precuneus, revealed a reliance on bottom-up (stimulus-driven) connections in AS participants that contrasted with a dominance of top-down (cognitive control) connections from prefrontal cortex observed in control participants. These results provide detailed support for previously hypothesised central disconnectivity in ASD and specify discrete brain network targets for diagnosis and therapy in ASD.

## Highlights

• We used MRI techniques to assess the connectivity in language networks in AS.
• Grey-matter heterogeneity of MR images correlated with volume of caudate in AS.
• Hence, caudate nuclei were used as seed ROIs in connectivity analyses: PPI, DCM.
• Grey-matter heterogeneity differently tuned caudate connectivity in AS, controls.
• DCM of language circuitry featured bottom-up models in AS and top-down in controls.

## 1 Introduction

Asperger syndrome (AS) is an Autism Spectrum Disorder (ASD) in which impairment in emotional and social interaction occurs in the context of relatively preserved development of general intellectual skills and verbal language. However, atypical language is often present as qualitative abnormalities in prosody, rate, volume, pragmatics or frequency of speech production (Volkmar and Klin, 2000).

The “Weak Central Coherence” hypothesis of ASD refers to a particular cognitive style characterised by a bias toward processing details rather than global aspects of information (Happé and Frith, 2006). This possible though not generalisable ASD profile, explains the superiority of some ASD individuals in the performance of visuospatial tasks compared to typically developed controls; likewise, it broadly suggests reduced top-down (cognitive) regulation and increased bottom-up (stimulus-driven) information processing (Happé and Frith, 2006). Correspondingly, at the neural level, individuals with ASD show aberrant functional connectivity between distributed cortical regions (Just et al., 2004; Schipul et al., 2011) and a relative over-development of short compared to long-range cortical structural connections (Casanova and Trippe, 2009). The nature and pattern of the interregional disconnectivity proposed to underlie these observations are consistent with genetic and environmental determinants occurring early during neurodevelopment (Geschwind, 2008; Kana et al., 2011).

Neurodevelopmental events like myelination, axonal growth and synaptic remodelling shape the pattern of connectivity and putatively contribute to cortical complex morphology (e.g. cortical folding) (Mangin et al., 2010). Accordingly, cortical surface features including folding are regarded as an indirect expression of the balance between local and long-range connectivity, arising in part from axonal tension (Hilgetag and Barbas, 2006; Van Essen, 1997).

In AS and ASD, MRI cortical surface abnormalities are documented: for instance, Nordahl et al. (2007) examined the cortical folding and found deeper intraparietal sulci in AS relative to typically developed control individuals. In addition, Wallace et al. (2010) reported cortical thinning in temporal, parietal and frontal regions in ASD compared with controls. In ASD, MRI has been used to characterise structural (DTI and volumetric) (e.g. Barnea-Goraly et al., 2004; Langen et al., 2007, 2012) and functional (e.g. Just et al., 2004; Schipul et al., 2011) correlates of local and inter-regional disrupted connectivity.

In a previous structural MRI study of Asperger syndrome (AS) (Radulescu et al., 2013), we demonstrated increased grey matter heterogeneity relative to controls using a surface-based method: texture analysis (Ganeshan et al., 2008). We also observed that whole brain measures of grey matter heterogeneity (measured with TA) correlated with the relative volume of the caudate nuclei in AS participants (Radulescu et al., 2013).

Texture analysis (TA) enables the complex characterisation of the gray-level pattern on MR images, the pixel inter-relations and spectral properties of the images (Kassner and Thornhill, 2010). By using automated algorithms, TA extracts quantitative features that evaluate the intensity and distribution of the signal on MR images (Castellano et al., 2004). Neuropsychiatric applications of MRI texture analysis include epilepsy (de Oliveira et al., 2013), mild traumatic brain injury (Holli et al., 2010), multiple sclerosis (Theocharakis et al., 2009), Attention Deficit Hyperactivity Disorder (ADHD) (Chang et al., 2012), Alzheimer disease (Zhang et al., 2012), and schizophrenia (Ganeshan et al., 2010). Although the relationship between MRI-derived textural parameters and brain architecture is still unclear, myelo- and cytoarchitectural features (the main contributors to the MRI T1 signal) may explain the grey-level ‘heterogeneity’ on MR images measured with TA (Annese, 2012; Eickhoff et al., 2005; Osechinskiy and Kruggel, 2009).

Based on theoretical relationships between complex cortical morphology and interregional connectivity, and reinforced by the findings of our previous studies, we hypothesised that subtle structural MRI abnormalities, expressed as increased grey matter heterogeneity and correlating with the morphometric integrity of basal ganglia (caudate nucleus), will relate to disrupted connectivity of cortico-striatal networks in AS during word production. In short, distributed grey matter abnormalities in AS reflect a process that compromises interaction between cortical and subcortical regions and ultimately communication skills in AS.

We focussed on the word production/verbal fluency paradigm in part due to the widely demonstrated role of cortico-striatal interaction in the control of speech production (Price, 2010). One proposed role of the caudate nucleus in word generation is in selecting between competing responses in a proficiency-dependent manner, as exemplified in the language-switching of multilingual individuals (Abutalebi et al., 2013; Garbin et al., 2011; Simard et al., 2013; Tan et al., 2011). Related to this mechanism is the proposal that caudate engagement may fine-tune interactions between automatic and more complex language processing (Friederici, 2006), for example during rule-based inflection of regular verbs, (Oh et al., 2011) or the resolution of word ambiguity (Ketteler et al., 2008). These accounts of basal ganglia contributions to language also fit with a proposal that, for general executive and motor functions, the striatum modulates thalamo-cortical information flow by inhibiting unwanted competing information, thereby enhancing the desired behavioural responses. This may be achieved in part via a dopaminergic regulatory mechanism originating in the substantia nigra (Murty et al., 2011). In people with AS and other ASD, disruption of cortico-striatal contributions to cognitive control may foster the expression of repetitive restricted behaviours including stereotyped language, abnormal intonation or prosody and emotionally/socially impoverished conversations (Pina-Camacho et al., 2012). Correspondingly, the basal ganglia (including caudate nucleus and lentiform nucleus) and their cortical connections are structurally affected in ASD and implicated in stereotypical behaviours and executive dysfunction (Langen et al., 2007; Stanfield et al., 2008; Turner et al., 2006).

**Fig. 1:**
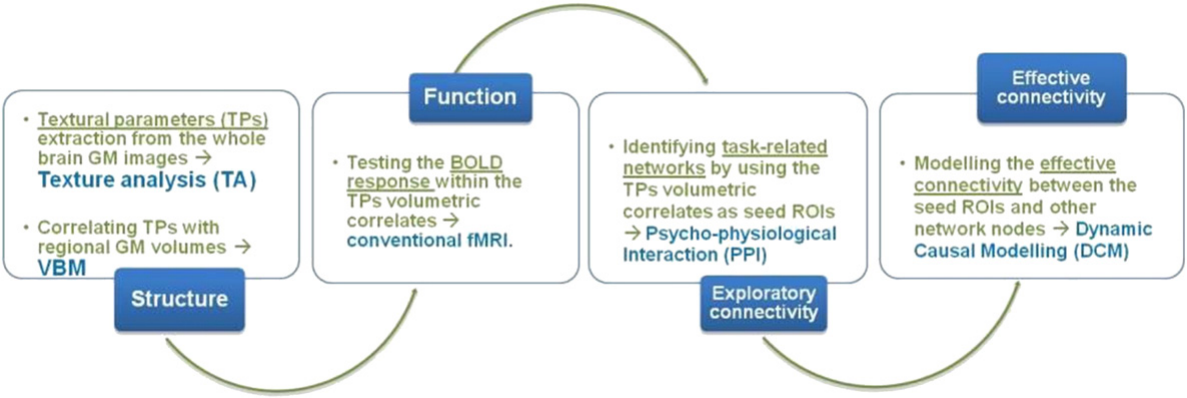
Integrative “stepwise” strategy for linking distributed MRI structural abnormalities to brain regions functionality and inter-connectivity in Asperger syndrome.

To test our hypothesis that, in AS, the observed grey matter heterogeneity associated with the integrity of basal ganglia (specifically caudate nucleus) relates to disconnectivity of cortico-striatal networks during speech production, we applied an integrative “stepwise” strategy combining complex structural and functional MRI datasets within the same participants (Fig. 1):

1. In a first step, we extracted textural parameters for characterizing grey matter heterogeneity on MR images. We compared texture parameters between AS participants and controls and found regional volumetric correlates of texture parameters (for example, the grey matter volume of caudate nuclei; Radulescu et al., 2013).
2. We then measured, using fMRI, regional brain responses evoked during a verbal fluency task that elicits activation in basal ganglia/caudate. We tested for experimental condition and group effects on the caudate task-evoked activity.
3. Subsequently, we identified cortico-caudate networks during word production using functional connectivity analyses (psycho-physiological interaction; PPI). We thereby identified caudate connections that were differently modulated by grey matter textural parameters in AS and controls (diagnosis-by-texture parameter interaction on PPI contrast images).
4. We further characterised the effective connectivity of cortico-caudate networks using dynamic causal modelling (DCM). We therefore modelled intrinsic (task-independent) and modulatory (task-dependent) connections between caudate, and PPI identified nodes in AS and controls.

## 2 Method

### 2.1 Participants

Forty-eight adult participants representing 22 individuals with AS diagnosis (11 males) and 26 controls (12 males) were included in the study. All participants gave written informed consent in accordance with the Declaration of Helsinki (1991), and the procedures were approved by the National Hospital for Neurology and Neurosurgery (NHNN) Research Ethics Committee. Functional brain responses evoked during a verbal fluency task were combined with textural information from segmented grey matter structural MR images. Data from this sample are reported in published studies (Beacher et al., 2012a, 2012b; Radulescu et al., 2013). Inclusion and exclusion criteria are presented in these papers. In brief, no significant differences between groups were observed for age, handedness or verbal fluency (number of words produced during the word generation task). However, a significant difference in the National Adult Reading Test (NART) score was observed (AS < controls; mean AS = 29.95 ± 7.99, mean controls = 35.08 ± 6.25, F(1,46) = 6.02, p = 0.016). Likewise, ten participants had maintenance treatment with antidepressants (SSRIs) (Table 1).

### 2.2 Functional imaging during the verbal fluency paradigm

The fMRI protocol for the verbal fluency task is described in an earlier paper (Beacher et al., 2012a). Briefly, echo-planar images (TR = 2.4 sec, TE = 50 ms, 5 mm slice thickness, 25 slices) were acquired on a 1.5 T Siemens Avanto scanner (Siemens Medical Systems AG, Erlangen, Germany) with 145 volumes collected per participant. The verbal fluency paradigm was created as a response-locked design, comprising six 40-second word generation conditions (letters T, L, B, O, A, N) and six 40-second control conditions during which participants responded with button presses to briefly presented visual stimuli (flashing crosses). Functional images were spatially realigned, motion corrected, normalised to the MNI (Montreal Neurological Institute) template then smoothed with an 8 mm Gaussian kernel using SPM8 (Wellcome Trust Centre for Neuroimaging; http://www.fil.ion.ucl.ac.uk/spm/software/spm8/). Pre-processed images were then analysed within General Linear Models (GLM) including regressors for the ‘letter’ condition composed of the time onsets when the participant produced an appropriate word and the ‘control’ condition composed of the time onsets when the participant saw the flashing crosses. Importantly, time onsets of the visual stimuli in the ‘control’ condition were matched to the onsets of word production in the previous ‘letter’ block. Instruction blocks were also entered in the model. Each regressor was convolved with a canonical hemodynamic response function (HRF) together with time and dispersion derivatives. Motion parameters were included as covariates of no interest. Appropriate T contrasts were created for the ‘letter’ and ‘control’ conditions for second level (group) and connectivity analyses.

#### 2.2.1 Second-level (group) analysis of fMRI verbal fluency data

The individual contrast images for ‘letter’ and ‘control’ conditions were used as dependent variables in a flexible factorial General Linear Model (GLM) implemented in SPM8. Three factors were used as independent regressors: a ‘subject’ factor modelling between-subjects variance; a within-subjects factor (‘condition’) with two levels: ‘letter’ and ‘control’; a between-subjects ‘group’ factor testing for differences between AS and controls. Non-sphericity corrections were applied to account for violations of independence of errors and equality of variance for the within-subjects ‘condition’, respectively for the ‘subject’ factor. NART was introduced as a covariate in the GLM to control for possible effects on brain activation, especially in the language areas. Based on the GLM we estimated the main effects for ‘condition’ and ‘group’ and the interaction effect ‘condition’ by ‘group’.

In addition, we performed post hoc analyses within each group to better identify diagnosis specific brain activity during ‘letter’ and ‘control’ condition. Initially, we performed a conjunction analysis to find regions activated by both groups. We thereafter saved the corresponding conjunction masks at a very lenient threshold (p = 0.05 uncorrected). In the post hoc analyses, we tested for group-specific activations for the above condition contrasts while masking out the respective activity common to both groups. Further, we examined the correlation between brain activity and performance (number of words produced during ‘letter’). For this, if we found group-specific significant neural responses during the ‘letter’ condition, we saved these clusters as regions of interest (ROIs). We then extracted for each participant the mean of the ‘letter’ contrast image at the specified ROI (by using REX (http://gablab.mit.edu/index.php/research/95-gablab-site/gablab/people/swg) and performed a bivariate correlation between the extracted data and the performance (number of words).

For statistical inference, we used the topological False Discovery Rate (FDR) correction for multiple comparisons implemented in SPM8 (Chumbley et al., 2010). We report below the clusters surviving a corrected significance threshold of p < 0.05.

### 2.3 Texture analysis of grey matter images and VBM analysis based on textural parameters

In a previous study, textural parameters (TPs) (mean of grey level, entropy, uniformity) were extracted from the segmented grey matter images (Radulescu et al., 2013). Quantification of the texture parameters within the whole brain was performed on unfiltered and filtered grey matter images. For filtered data, three types of filters were applied: fine (filter width: 0.5 mm), medium (filter width: 1 mm), coarse (filter width: 1.5 mm). A set of texture parameters that differed between AS and controls was reduced to independent non-collinear variables through Principal Component Analysis (PCA), and subsequently used as regressors in a voxel-based morphometry (VBM) analysis on the grey matter segmented, modulated, normalised and smoothed MR images (Beacher et al., 2012b; Radulescu et al., 2013). One TP regressor (the PCA regression score representing a combination between filtered entropies and uniformities) correlated with the volume of caudate nuclei (Radulescu et al., 2013). This regression score was used in the present study to explore the relationship of grey matter textural heterogeneity with the caudate connectivity (see below).

### 2.4 Effective connectivity analyses

After creating subject-specific standard psycho-physiological interaction terms (PPIs) as measures of caudate connectivity, we investigated the texture parameters effect on the caudate connectivity at group-level, respectively we tested the specific hypothesis that grey matter textural abnormalities differently modulate caudate connectivity during verbal fluency in participants with Asperger syndrome (AS) compared with controls.

In addition, we performed a group-level analysis based on PPI contrast images to identify brain regions showing effective functional connectivity with the caudate nuclei during word generation independent of grey matter texture heterogeneity.

Finally, dynamic causal modelling (DCM) was used to test causal interactions within cortico-caudate networks, with ‘Bayesian model selection’ (BMS) and ‘Bayesian model averaging’ (BMA) applied to estimate and compare DC models that encompassed backward and forward connections between top-down cognitive control centres and sources of bottom-up informational input into word production.

#### 2.4.1 Psychophysiological interaction (PPI)

PPI for each participant was performed on the right and left caudate nuclei using standard SPM protocols. Details about the individual PPI analysis are described in the Supplementary method. Selection of the caudate nuclei as seed/source regions was predicated on the previously demonstrated effect of grey matter texture parameters on bilateral caudate volume (Radulescu et al., 2013). In addition, conventional analyses of our fMRI data revealed activation within caudate nuclei during word generation (see Results section and Supplementary Fig. 3), consistent with the previously demonstrated role of these structures in speech production (Price, 2010).

PPI for each participant was performed on the right and left caudate nuclei using standard SPM protocols. Details about the individual PPI analysis are described in the Supplementary method. Selection of the caudate nuclei as seed/source regions was predicated on the previously demonstrated effect of grey matter texture parameters on bilateral caudate volume (Radulescu et al., 2013). In addition, conventional analyses of our fMRI data revealed activation within caudate nuclei during word generation (see Results section and Supplementary Fig. 3), consistent with the previously demonstrated role of these structures in speech production (Price, 2010).

#### 2.4.2 Second-level (group) analysis based on PPI contrast images

Initially, we explored whether experimental manipulation (‘letter’ vs. ‘control’) and grey matter texture heterogeneity differently modulated caudate connectivity with other brain regions in AS and controls. Accordingly, we performed a second-level random effects (GLM) analysis with the individual PPI contrast images (‘letter’ vs. ‘control’) as dependent variables, diagnosis as group factor and grey matter texture and age as covariates. Grey matter texture covariate was the PCA regression score that predicted the caudate grey matter volume in the previous structural imaging analysis (Radulescu et al., 2013) (see above). Specifically, we tested for a diagnosis × grey matter texture score interaction that if significant, would have represented a differential modulatory effect of grey matter texture heterogeneity on the caudate connectivity in AS and controls. In addition, we examined the caudate connectivity during word generation vs. ‘control’ independent of grey matter texture heterogeneity (second-level analysis on PPI contrast images without texture score as covariate).

The statistical inference threshold was set for p < 0.05 after correction for multiple comparisons at cluster-level (FDR topological multiple comparisons correction).

#### 2.4.3 Dynamic causal modelling (DCM)

After identifying the key regions whose connectivity with the caudate nuclei is modulated by the grey matter texture parameters, we used this information to specify and test more complex models of effective connectivity (dynamic causal models — DCMs). We performed this analysis with DCM10 software implemented in SPM8 (http://www.fil.ion.ucl.ac.uk/spm/).

Like in the PPI analysis, we employed the standard protocol for DCM: time series extraction from the regions of interest, specifying for each participant a DCM adapted design matrix describing the GLM of the experimental paradigm (verbal fluency in our case), defining and estimating the DCMs, model inference by comparing the DCMs within the two groups with Bayesian Model Averaging (BMA) (Penny et al., 2010). Details about the first-level (participant-specific) GLM adapted for DCM are provided in the Supplementary method.

##### 2.4.3.1 Time series extraction

We specified four regions of interest (ROIs), the left inferior frontal gyrus, the right inferior frontal gyrus extending into the adjacent right anterior insula, bilateral caudate head, and bilateral precuneus. Our selection of ROIs was motivated by: a) PPI results that showed diagnosis differences in regression slopes mediated by grey matter textural properties; b) GLM second level/group analysis that demonstrated condition and diagnosis effects on specified ROIs' activations; and c) previously demonstrated activation during language production (Price, 2010) and visual attention tasks (Corbetta, 1998).

The summary of time series (the first eigenvariate) from all the voxels within the region of interest was extracted from F contrasts of the word generation condition (‘letter’) and visual attention (‘control’) condition.

The search of activated voxels within the ROI was guided for each individual by WFU Pickatlas (http://fmri.wfubmc.edu/software/PickAtlas) masks and group analysis results. The WFU masks comprised the left inferior frontal gyrus (L-IFG) pars opercularis and pars triangularis, the bilateral caudate head (dilation = 1 mm), the right inferior frontal gyrus/insula (R-IFG/Ins) and the bilateral precuneus (PCu). We held no a priori expectation of hemispheric differences in caudate or precuneus responses during word generation; bilateral involvement of caudate head is observed during language (Price, 2010) and other cognitive processing (i.e. attention switching; van Schouwenburg et al., 2010). Additionally, variability in activation peak locations across participants (≈ 10 mm) may obscure hemispheric differences in midline regions like the precuneus (Eickhoff et al., 2009a). Consequently, for creating simpler DCMs with maximum four nodes, we used bilateral masks for caudate and precuneus time-series extraction during word generation.

Details about participant-specific dynamic causal models construction are provided in the Supplementary method and Supplementary Table 1.

Details about participant-specific dynamic causal models construction are provided in the Supplementary method and Supplementary Table 1.

##### 2.4.3.2 DCM analysis at group level

**Fig. 2:**
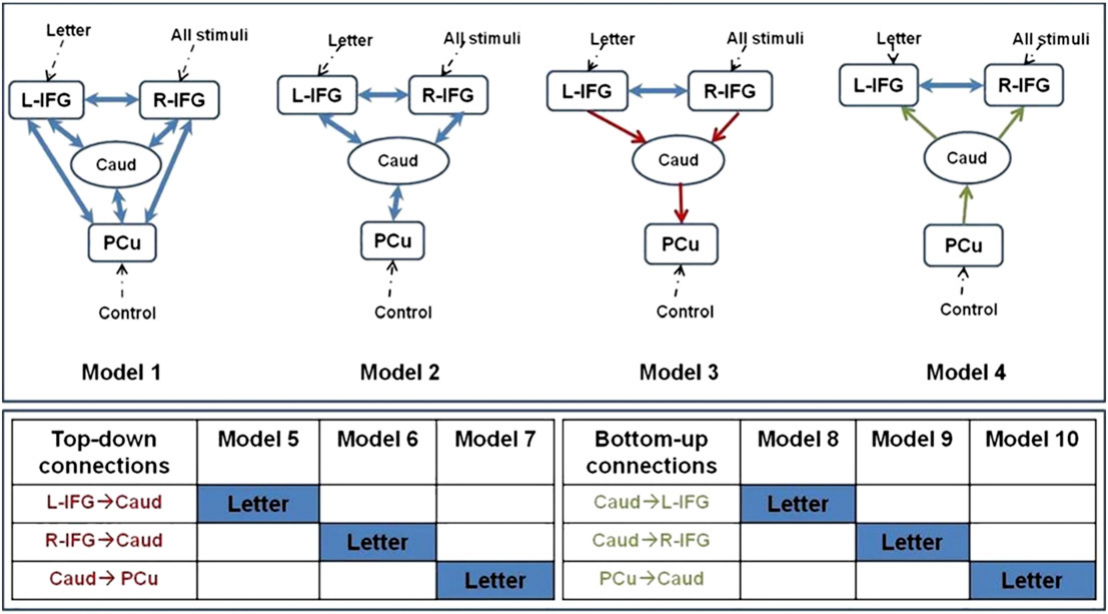
The architecture of the dynamic causal models (DCMs). *Upper panel*: The first DCMs featuring intrinsic connectivity independent of the experimental effects; backbone of the models represented by connections between four nodes: L-IFG, R-IFG, Caud and PCu. Caudate is represented as a relay between the frontal (L-IFG, R-IFG) and parietal (PCu) regions. The first two models feature only bi-directional connections (blue arrows); model 3 describes the top-down/backward connections (L-IFG, respectively R-IFG to Caud); model 4 describes the bottom-up/forward connections (PCu to Caud, Caud to L- and R-IFG). Stimulus-bound perturbations (driving inputs) entered the model in the frontal and parietal nodes (‘Letter’ through the L-IFG, ‘All stimuli’ through the R-IFG and ‘Control’ through PCu; black dashed arrows). *Bottom panel*: The modulatory effects of the condition of interest (‘Letter’-word production) represented in six DCMs — three based on the top-down/backward model 3 (left) and three based on the bottom-up/forward model 4 (right). ‘Letter’ condition modulates the connectivity strength of each top-down/bottom-up connection (blue cells). *Abbreviations*: L-IFG = left inferior frontal gyrus/insula; R-IFG = right inferior frontal gyrus/insula; Caud = caudate; PCu = precuneus.

I. Initially, we tested four DCMs (Fig. 2 upper panel) specified under the theory of “weak central coherence” in autism spectrum conditions (Happé and Frith, 2006). Specifically, we tested the hypothesis that while performing the experimental paradigm, AS individuals will favour the bottom-up (forward) connections, whereas the controls will merely rely on top-down (backward) connections.
II. Secondly, we tested models comprising modulatory effects of experimental manipulation on intrinsic connectivity (Fig. 2 bottom panel).

We performed a BMA separately on the first four models (no modulatory effects), followed by a BMA on the six models featuring ‘letter’ modulatory effects. In addition, the BMA was applied separately within each group.

BMA is an alternative approach to Bayesian model selection (BMS), suitable when groups represent different populations (in our case normative population and AS) (Stephan et al., 2010). This method performs the parameters' inference on the entire model space, not on a chosen model. BMA calculates each weighted averages of each model parameter (weighting giving by the posterior probability of each model) (Penny et al., 2010; Stephan et al., 2010).

Results reported here are the averaging results with an Occam's window defined by a minimal posterior odds ratio of π = 1/20, as previously used (Penny et al., 2006). In addition, we used the default number of samples for averaging parameters (n = 10,000). Of note, the “RFX” model averaging the Occam's windowing is specific to each participant. Consequently, each participant can have a different number of models in Occam's window (Penny et al., 2010).

## 3 Results

### 3.1 Conventional fMRI results showing brain activation during word production

**Supplementary Fig. 1:**
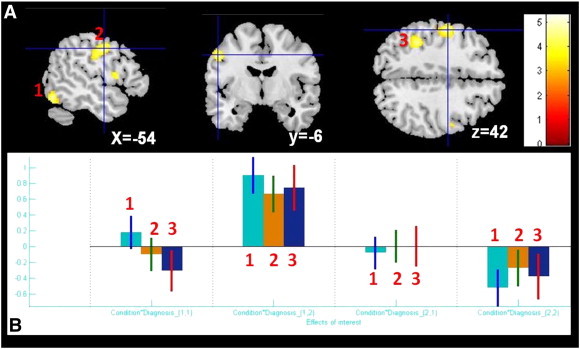
Results of conventional fMRI analysis (group-level GLM) during verbal fluency. A. Interaction effect condition × diagnosis showing significant BOLD response within three clusters (numbers 1–3 in red): 1. Left middle occipital gyrus (BA 37) (MNI: − 56 − 66 − 12; k = 145), 2. Left precentral gyrus (MNI: − 52 − 6 42; k = 283), 3. Left inferior parietal lobule (MNI: − 38 − 40 42; k = 247). B. Plots of effect size and directionality created with REX (http://gablab.mit.edu/index.php/research/95-gablabsite/ablab/people/swg). Condition 1 = „letter„, condition 2 = „control„, diagnosis 1 = controls, diagnosis 2 = AS.

Whole-brain analysis showed a condition effect (‘letter’ vs. ‘control’) as previously described (Beacher et al., 2012a). We also found group differences qualified by an interaction condition × diagnosis effect in the left middle occipital gyrus (BA 37), left precentral and the left inferior parietal lobule (Supplementary Fig. 1). Of note, two of these regions (L-BA 37 and the left inferior parietal lobule) showed the significant diagnosis effect (AS > controls) in our second-level analysis/full factorial model based on individual contrasts ‘*letter*’ > ‘*control*’ (Beacher et al., 2012a).

Whole-brain analysis showed a condition effect (‘letter’ vs. ‘control’) as previously described (Beacher et al., 2012a). We also found group differences qualified by an interaction condition × diagnosis effect in the left middle occipital gyrus (BA 37), left precentral and the left inferior parietal lobule (Supplementary Fig. 1). Of note, two of these regions (L-BA 37 and the left inferior parietal lobule) showed the significant diagnosis effect (AS > controls) in our second-level analysis/full factorial model based on individual contrasts ‘*letter*’ > ‘*control*’ (Beacher et al., 2012a).

**Supplementary Fig. 2:**
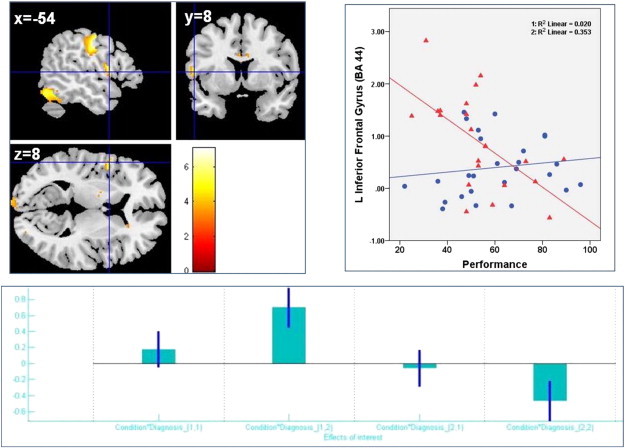
Conventional fMRI post hoc analysis results (within group specific effects). Top left panel: statistical parametric map showing a left inferior frontal gyrus (L-IFG) cluster (BA 44) with higher activation in AS group during “letter” > “control”. Top right panel: plot showing significant correlation between the mean extracted from the L-IFG cluster and performance during word production in AS (1:R^2^ = R squared in controls; 2:R^2^ = R squared in AS; blue slope and circles = controls; red slope and triangles = AS). Bottom panel: Plots of effect size and directionality created with REX (http://gablab.mit.edu/index.php/research/95-gablabsite/gablab/people/swg). Condition 1 = „letter„, condition 2 = „control„, diagnosis 1 = controls, diagnosis 2 = AS.

In the post hoc analyses exploring the brain activation specific for each group, the AS sample additionally engaged a cluster within the left inferior frontal gyrus (BA 44) (MNI: − 54 8 8; k = 12; p = 2.2250e-008; FDR topological correction) during ‘letter’ vs. ‘control’ conditions. The results of post hoc analyses are presented in the ‘Supplementary Fig. 2’.

In the post hoc analyses exploring the brain activation specific for each group, the AS sample additionally engaged a cluster within the left inferior frontal gyrus (BA 44) (MNI: − 54 8 8; k = 12; p = 2.2250e-008; FDR topological correction) during ‘letter’ vs. ‘control’ conditions. The results of post hoc analyses are presented in the ‘Supplementary Fig. 2’.

The mean signal change extracted from the left inferior frontal cluster also correlated with the performance during the word generation in the AS group (Supplementary Fig. 2).

The mean signal change extracted from the left inferior frontal cluster also correlated with the performance during the word generation in the AS group (Supplementary Fig. 2).

**Supplementary Fig. 3:**
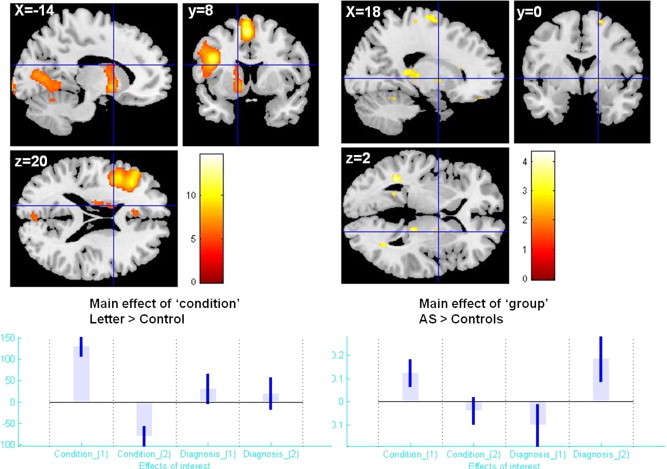
Results of conventional fMRI analysis (group-level GLM) during verbal fluency showing caudate activity. Left column: main effect of condition (“letter” > “control”) showing strong BOLD response in a left basal ganglia cluster comprising peaks within caudate, putamen, and pallidum. Right column: non-significant main effect of diagnosis 40 (AS > controls) on a right pallidum cluster. Statistical parametric maps overlaid on a MNI template. Plots of effect size created with REX (http://gablab.mit.edu/index.php/research/95-gablab-site/gablab/people/swg).

Specifically for the basal ganglia functionality during the word generation, we found a significant main effect of ‘condition’ for the contrast ‘letter’ > ‘control’ in a cluster comprising left pallidum, left caudate nucleus and left putamen (MNI coordinates: − 12 6 − 4, − 18 − 12 22, respectively − 16 2 12; k = 394; p = 1.3659e-011; FDR topological correction) (Supplementary Fig. 3).

Specifically for the basal ganglia functionality during the word generation, we found a significant main effect of ‘condition’ for the contrast ‘letter’ > ‘control’ in a cluster comprising left pallidum, left caudate nucleus and left putamen (MNI coordinates: − 12 6 − 4, − 18 − 12 22, respectively − 16 2 12; k = 394; p = 1.3659e-011; FDR topological correction) (Supplementary Fig. 3).

We observed a main effect of ‘group’ (diagnosis) for the contrast AS > controls in a basal ganglia cluster centred on right Pallidum (MNI coordinates 18 0 2; k = 14), but which did not survive to the multiple comparisons correction FDR at cluster level (Supplementary Fig. 3). There were no significant interaction effects ‘condition’ ×  ‘diagnosis’ within the basal ganglia that survived the multiple comparison corrections at cluster level. We also tested for NART effects on brain activation during the word production. There were no significant results specific to our regions of interest.

We observed a main effect of ‘group’ (diagnosis) for the contrast AS > controls in a basal ganglia cluster centred on right Pallidum (MNI coordinates 18 0 2; k = 14), but which did not survive to the multiple comparisons correction FDR at cluster level (Supplementary Fig. 3). There were no significant interaction effects ‘condition’ ×  ‘diagnosis’ within the basal ganglia that survived the multiple comparison corrections at cluster level. We also tested for NART effects on brain activation during the word production. There were no significant results specific to our regions of interest.

### 3.2 PPI connectivity results

#### 3.2.1 Influence of grey matter heterogeneity on caudate connectivity during verbal fluency

Inclusion of the grey matter texture score within the PPI group analysis demonstrated an interaction diagnosis × texture effect, indicating covariate-dependent heterogeneity of regression slopes in AS and controls, when regressing all the voxels in the brain over the individual PPI contrast images. We re-iterate that PPI contrast images represented the task-modulated (‘letter’ vs. ‘control’) and participant-specific caudate connectivity with target regions elsewhere in the brain.

**Fig. 3:**
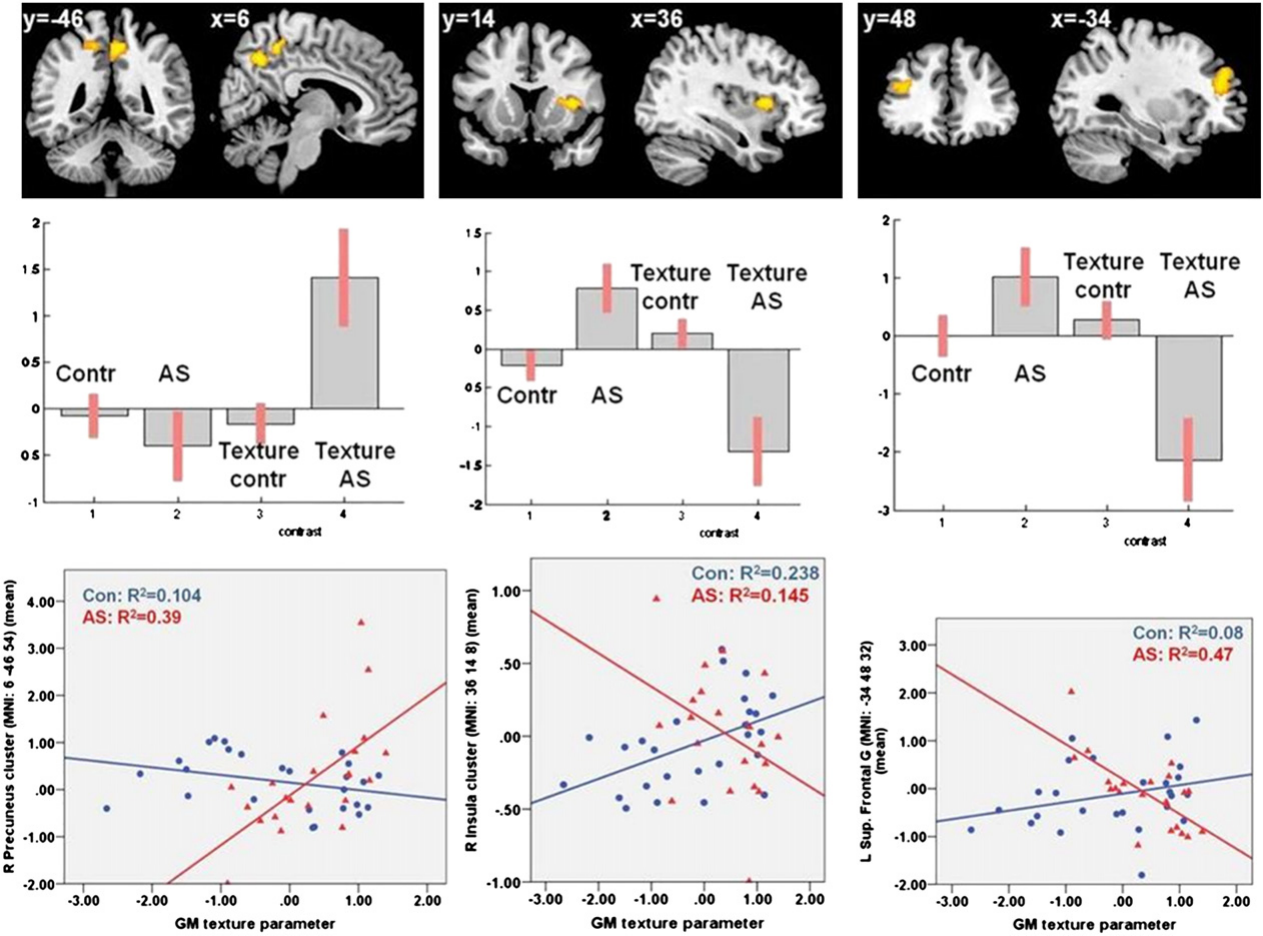
*PPI analysis*: interaction ‘diagnosis × texture score’ on the caudate connectivity. The top panels show statistical parametric maps overlaid on an MNI template. The middle and bottom panels show the plots (effect size and correlation) representing the texture effect on the left and right caudate connectivity. *Left*: in AS there is a positive relationship between texture score and the connectivity between the left caudate and right precuneus during word generation. *Middle*: in controls there is a positive task-dependent correlation between the texture score and the connectivity between left caudate and the right inferior frontal gyrus/insula, whereas in AS the reverse is observed. *Right*: in AS there is a negative correlation between the texture score and the connectivity between the right caudate and the left superior frontal gyrus during word production vs. visual attention. *Abbreviations*: R^2^ = the square of coefficient of multiple correlation; Con/Contr = controls; AS = Asperger syndrome; GM = grey matter; R = right; L = left; G = gyrus; MNI = Montreal Neurological Institute.

In other words, the texture score modulated the type of connectivity between caudate and other brain regions as a function of diagnosis. Specifically, the texture score in control participants positively predicted the strength of connectivity between left caudate and right anterior insula, whereas the texture score in AS demonstrated an inverse relationship with the connectivity strength between the same two regions (MNI: 36 14 8; k = 240 voxels; p = 1.0543e-004) (Fig. 3 middle panel).

**Supplementary Fig. 4:**
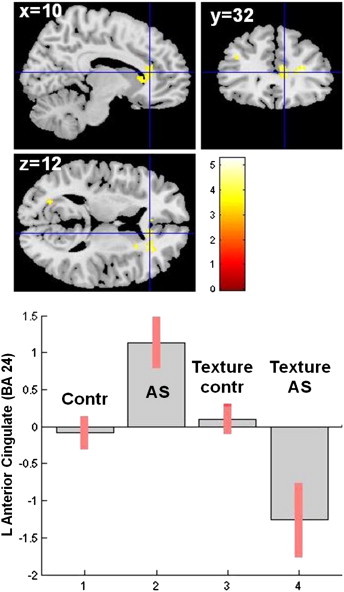
PPI analysis: interaction ‘diagnosis × texture score’ on the caudate–anterior cingulate cortex (ACC) connectivity. Top panel: statistical parametric map showing the location (MNI coordinates) of the ACC cluster. Bottom panel: plot (effect size) representing the texture effect on the left caudate–right ACC connectivity. *Abbreviations*: Contr = controls; AS = Asperger syndrome; L = left; MNI = Montreal Neurological Institute.

Interestingly, a similar though statistically weaker pattern of connectivity changes was also observed between the left caudate and right anterior cingulate cortex (ACC) BA 24 (MNI: 10 32 12; alpha = 0.051 FDR topologically corrected for multiple comparisons; p = 0.0046; k = 108 voxels) (Supplementary Fig. 4).

Interestingly, a similar though statistically weaker pattern of connectivity changes was also observed between the left caudate and right anterior cingulate cortex (ACC) BA 24 (MNI: 10 32 12; alpha = 0.051 FDR topologically corrected for multiple comparisons; p = 0.0046; k = 108 voxels) (Supplementary Fig. 4).

Effects in the opposite direction (positive correlation in AS, negative in controls) were observed for the connectivity between left caudate to right precuneus (MNI: 6 − 46 54; k = 476 voxels; p = 5.2718e-007) (Fig. 3 left panel). An interaction between diagnosis and texture score (negative correlation in AS participants, no correlation in controls) was observed for the connectivity of the right caudate with the left superior frontal gyrus (MNI: − 34 48 32; k = 277 voxels; p = 2.9982e-005) (Fig. 3 right panel).

In summary, during verbal fluency, people with AS showed differences from controls in connectivity between caudate nucleus and cortical centres implicated in cognitive, emotional and social processes that could be attributed to distributed features of grey matter microstructure.

#### 3.2.2 Group differences in functional connectivity of the caudate nuclei not explained by the grey matter texture score

**Supplementary Fig. 5:**
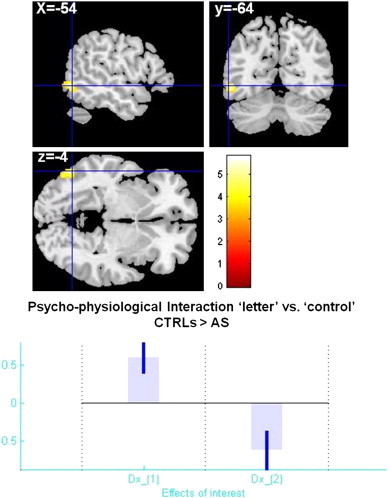
Results of PPI analysis at group-level independent of grey matter texture heterogeneity modulation. Manipulation of experimental condition (“letter”–word production vs. “control”–visual attention) positively modulated the right caudate coupling with the left middle temporal/fusiform gyrus in controls, whereas the opposite (negative modulation) was observed in Asperger syndrome (AS). Statistical parametric maps overlaid on a MNI template. Plots of effect size created with REX (http://gablab.mit.edu/index.php/research/95-gablab-site/gablab/people/swg).

In addition, PPI analysis showed verbal fluency task-related modulation (‘letter’ vs. ‘control’) between the right caudate and the left middle temporal gyrus (BA 37) (close to the Visual Word Form Area) (MNI: − 54 − 64 − 4; k = 151 voxels; p = 0.001 FDR corrected at cluster-level), positive in AS group and negative in controls (Supplementary Fig. 5).

In addition, PPI analysis showed verbal fluency task-related modulation (‘letter’ vs. ‘control’) between the right caudate and the left middle temporal gyrus (BA 37) (close to the Visual Word Form Area) (MNI: − 54 − 64 − 4; k = 151 voxels; p = 0.001 FDR corrected at cluster-level), positive in AS group and negative in controls (Supplementary Fig. 5).

### 3.3 DCM results

#### 3.3.1 Task-independent intrinsic causal connectivity

**Fig. 4:**
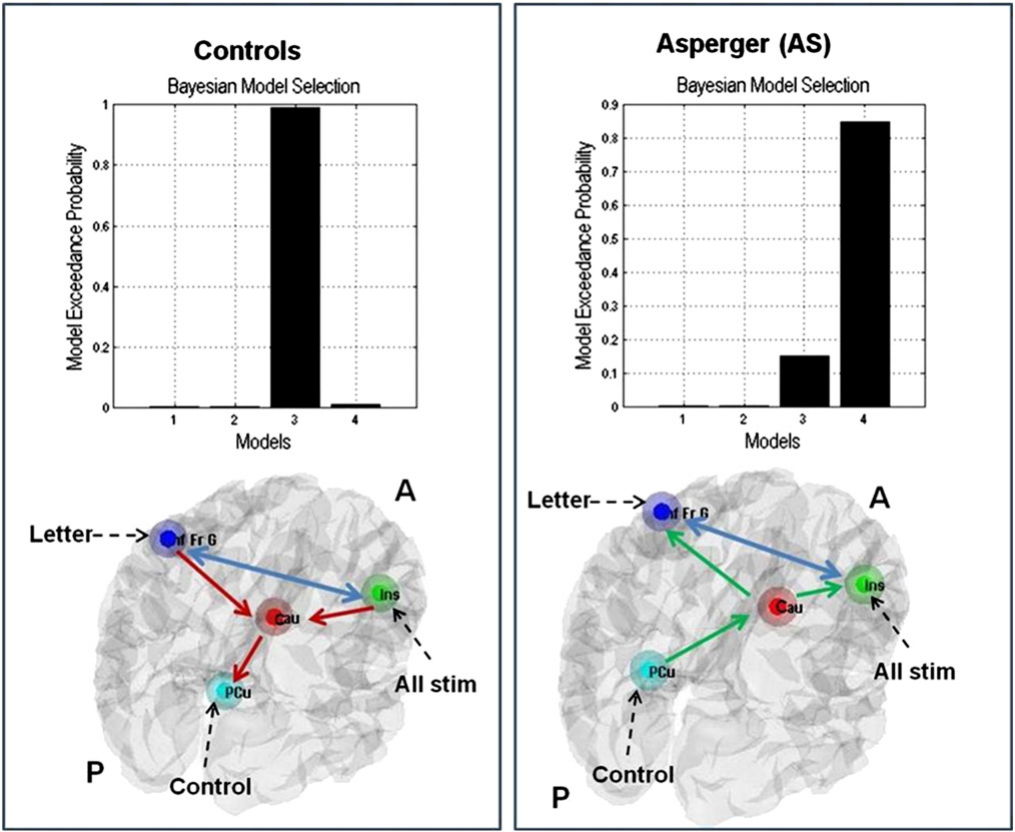
The results of the Bayesian model averaging (BMA) on the models featuring the intrinsic connectivity independent of experimental modulatory effects. In controls, a top-down/backward model is favoured (left), while in AS the bottom-up/forward model is predominant. *Legend*: Inf Fr G = inferior frontal gyrus; Ins = right inferior frontal gyrus/insula; Cau = caudate; PCu = precuneus; stim = stimuli.

In connectivity models incorporating prefrontal regions (confined to inferior frontal gyrus), precuneus and caudate nuclei, we first tested for group differences in connectivity independent of the language task. Across the first four specified models, the AS individuals demonstrated causal connectivity driven by bottom-up connections from precuneus to caudate nucleus and from caudate nucleus to left, and right IFG respectively (i.e. model 4 was favoured in this group). In contrast, data from controls (healthy volunteers) better fitted a model of descending causal connectivity (model 3), containing bidirectional connections between the left and right IFG and top-down connections from left IFG to caudate nucleus, right IFG to caudate and caudate to precuneus (Fig. 4).

#### 3.3.2 Causal connectivity during word generation

**Fig. 5:**
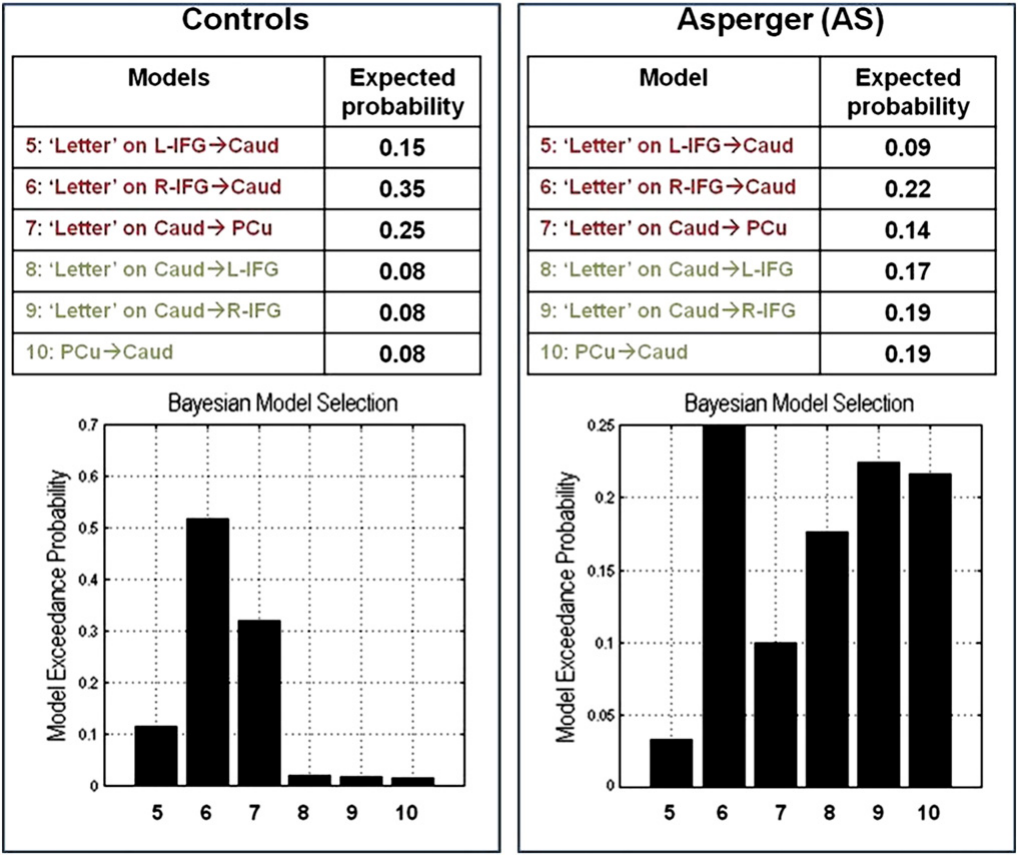
The results of the Bayesian model averaging (BMA) on the models featuring the experimental modulatory effects of ‘Letter’ on intrinsic connections. In controls, the top-down/backward models are the strongest (left), while in AS the bottom-up/forward models and top-down models have comparable weights. *Abbreviations*: L-IFG = inferior frontal gyrus; R-IFG = right inferior frontal gyrus/insula; Caud = caudate; PCu = precuneus.

In AS, word generation (‘letter effects’) evoked non-specific changes in interregional connectivity, such that five of the six models of task effects had comparable lower exceedance and expected probabilities (Fig. 5 right). Conversely, in controls, within the model space there was a trend for ‘letter’ effects to modulate exclusively top-down connections, mainly between R-IFG and caudate (Fig. 5 left).

Of note, model 5 describing the modulatory effect of ‘letter’ on the backward connectivity between the L-IFG (Broca's area) and the caudate revealed a low exceedance probability in both controls and AS participants. This pattern indicates that top-down modulation from L-IFG to caudate may not be related to the word production per se, but rather to another concomitant process (i.e. response selection and/or inhibition).

In summary, during verbal fluency, people with AS still favoured bottom-up ‘letter’ modulatory DCMs in addition to top-down models, whereas the controls exclusively favoured the top-down ‘letter’ modulatory DCMs.

## 4 Discussion

Our study set out to test the hypothesis that distributed MRI grey-matter (GM) heterogeneity previously correlated with the GM volume of the caudate nuclei in Asperger syndrome (AS) (Radulescu et al., 2013) also relates to abnormalities in the cortico-striatal networks during word production.

To test this hypothesis we applied a strategy that integrated in a stepwise manner results from several structural and functional (conventional and connectivity) MRI analyses. We had previously identified distributed anomalies in grey matter heterogeneity (a high-level textural measure related to microstructural differences) that correlated also to caudate volume in individuals with AS compared to controls (Radulescu et al., 2013).

Hereupon, we examined, during performance of a verbal fluency task, how the imputed heterogeneity of grey matter may influence differences between people with and without AS in the functional connectivity between caudate nucleus and cortical regions. Moreover, we incorporated caudate activity into dynamic causal models of connectivity to test for group differences in top-down versus bottom-up information processing suggested theoretically.

Two main findings stood out as significant for understanding Asperger syndrome pathogenesis:

1. The functional connectivity of the caudate nuclei was atypical in participants with AS relative to matched controls. Furthermore, subtle distributed MRI morphological abnormalities, measured with grey matter textural parameters, abnormally modulated connectivity between caudate nucleus and discrete cortical regions in AS.
2. Models of connectivity incorporating the caudate nucleus, and testing for causal influences of one region on another, showed greater dominance of bottom-up/feed-forward parieto-striato-frontal connections in AS. This contrasted with dominance of top-down/feedback fronto-striato-parietal functional connectivity in controls during speech production and visual attention.

Thus in the present study, we demonstrated that grey matter textural parameters in AS impact on the connectivity between the caudate nuclei and several frontal and parietal regions (i.e. right insula/IFG, ACC, precuneus, dorsolateral PFC) compared with control participants. Taking these grey matter textural measures as a high-level expression of distributed, developmentally determined differences in cytoarchitecture and local connectivity, our findings support the hypothesis that ASDs are disconnection syndromes. Thus, previous MRI studies attribute to the regions encompassed within the ‘texture-sensitive’ caudate networks high functional relevance to language, executive functions and selective visual attention (Cavanna and Trimble, 2006; Elliott, 2003; Gazzaley and Nobre, 2012; Price, 2010; Robbins, 2007).

The atypical connectivity between caudate and frontal language/cognitive control regions that we observed in AS extends earlier evidence from neuroimaging studies of sentence comprehension that show low functional connectivity along the language circuitry in autism compared with controls (Just et al., 2004; Kana et al., 2006). At a general level, our results converge with reports that show atypical fronto-striatal-parietal circuitry during cognitive control in ASDs (Kana et al., 2007; McAlonan et al., 2002; Takarae et al., 2007; Turner et al., 2006).

Interestingly, during word production, the AS group demonstrated a higher activation within a small region of Broca's area (left inferior frontal gyrus, BA 44) that was also inversely correlated with the performance (number of words produced during the ‘letter’ condition). This possibly reflects an inefficiency pattern related to dysfunction within the language circuitry. In a way analogous to neuroimaging findings that show genetic and age-related influences on dorso-lateral prefrontal cortex activation during executive function tasks (Sambataro et al., 2009), our results possibly indicate that AS individuals require a higher level of engagement of task-relevant areas to sustain a performance comparable to that of typically developed controls.

A novel observation is that of abnormal caudate–precuneus connectivity in Asperger syndrome expressed as a function of grey matter heterogeneity. This may relate to increased posterior cortical activation and local connectivity previously documented and suggested to subserve unusual visuospatial abilities present in some individuals with ASD (Minshew and Keller, 2010).

Alternatively, it may relate to a distinctive pattern of Default Mode Network (DMN) activity in AS. In healthy controls, variability in the DMN-caudate connectivity is sensitive to the genetic status (i.e. variability in dopaminergic genes) (Sambataro et al., 2013). In addition, children with ASD demonstrate reduced connectivity between precuneus and caudate nuclei during rest (Lynch et al., in press). The converging evidence supports the interpretation of that there is an imbalance in DMN activity in people with ASD, with reduced anterior-posterior connectivity at rest, in contrast to a quasi-normal connectivity in the task-positive network (Broyd et al., 2009). Moreover, the typical anticorrelation between task-negative and task-positive networks appears disrupted in ASD (Broyd et al., 2009). In this context, our PPI results, demonstrates a positive correlation between GM heterogeneity and caudate- precuneus connectivity in AS, and hence may reflect “underconnectivity” within the DMN in this disorder. However, a cautionary note is necessary with respect to the concept of “underconnectivity”: DMN connectivity may be confounded by factors including age/developmental status, and structural heterogeneity (Lynch et al., in press).

In addition, the PPI analysis with the caudate nuclei as seed regions demonstrated a main effect of diagnosis unexplained by the texture score: AS participants, relative to controls, showed a negative verbal fluency task modulation of the coupling between the right caudate and the left middle temporal/fusiform gyrus. Interestingly, we had previously noted a strong main effect of diagnosis within the same left fusiform region, which was activated to a much greater extent in AS compared to control participants (Beacher et al., 2012a). This region corresponds to the visual word form area (VWFA) (Dehaene et al., 2002; McCandliss et al., 2003), suggesting that orthographic representations are more relevant to word generation in Asperger syndrome. While this explanation seems plausible, it should be also stated that the same area shows reliable activation during fMRI paradigms unrelated to reading and orthographic representation of language (including viewing and naming pictures, naming colours, action decision to nonobjects, repeating and thinking about the meaning of heard words) (Price and Devlin, 2003). This indicates that VWFA hyperactivation in our AS group may reflect a different role in word production that deserves further exploration.

In general, our results are interpretable in the context of previously demonstrated brain anterior-posterior underconnectivity in ASD (Just et al., 2004; Schipul et al., 2011). Possibly related to weaker connectivity, occipital regions implicated in reading apparently activate quasi-independently and escape the fronto-striatal mediated top-down control. Ultimately, this may lead to a less efficient strategy in word production. One expression of this may be the attenuated cognitive flexibility and slower processing widely observed in ASD when performance occurs in the context of time-constraints or complex cognitive demands (Kana et al., 2011). From this perspective, the additional recruitment of the VWFA by the AS group may represent a persistent, unregulated and unnecessary engagement of orthographic representations (here visualisation of the target letter during the ‘letter’ condition). Another interpretation relates to the basal ganglia roles as key nodes in the prefrontal–striatal circuits, which mediate attention and response set switching, probably through a mechanism of salience-based selective sensory gating (Gray et al., 2013; van Schouwenburg et al., 2010).

In the present study we undertook further model-based connectivity analyses to gain more insight into the impact of ASDs causal interactions between brain regions engaged by the language (word generation) task and its control condition. We used dynamic causal modelling (DCM) first to explore the directionality along the intrinsic connectivity independent of direct task effects. In AS, the model that best accounted for the temporal dynamics of interregional activations emphasised feed-forward connections from the precuneus to caudate and then onto the left IFG and right IFG/insula. This stood out as quite different from the optimal model in controls, which was characterised by top-down backward connections from the left IFG and right IFG/insula onto caudate and from the caudate to precuneus.

These causal models confirmed the prediction that there is dominance within Asperger syndrome (AS) of bottom-up influences from the posterior regions supporting stimulus-driven visual strategies during cognitive processes (Kana et al., 2006). In contrast, stronger top-down connections in controls are suggestive of a preferential use of strategies based on executive functions/cognitive control during task execution. Moreover, these findings of bottom-up directionality in ASD fit well with the ‘weaker central coherence theory’ that explains various autism symptoms by a reduced top-down control leading to a deficiency in complex information integration (Happé and Frith, 2006; Minshew et al., 1997).

Functional maturation of language circuits is linked to the development of effective top-down control: children engage a similar but wider network compared with adults, and rely to a greater extent on the bottom-up processing (Bitan et al., 2006, 2009). From this perspective, our results in AS subjects possibly reflect a ‘dysmaturation’ of the language processing circuits. Interestingly though, the causal connectivity analyses that explicitly incorporated modulatory effects of the language task did not profoundly alter these connectivity patterns. In participants with AS, word generation, prompted by presentation of letter stimuli, had only a weak impact on prefrontal-caudate connectivity. However, in the controls prevailed the model of connectivity between right IFG/insula and caudate, confirming additional executive/cognitive control contributions to word generation in this group. The right IFG/insula and striatum participate in complex lexical processing (i.e. motor aspects of speech production and planning, monitoring of semantic decisions, resolving lexical ambiguities, sequencing, prosody detection) (Chan et al., 2013; Eickhoff et al., 2009b; Friederici, 2006; Ketteler et al., 2008; Oh et al., 2011; Price, 2010; Rota et al., 2010; van de Ven et al., 2009; Whitney et al., 2009). Likewise, a more general contribution of these two regions to the cognitive control (selective attention, response selection, response inhibition and task switching) is also recognised (Hedden and Gabrieli, 2010; Jahfari et al., 2011; Robbins, 2007; Robinson et al., 2012). Thus, we interpret the differential modulatory effects of the language task in AS and controls as further evidence of atypical connectivity of cognitive control networks in autism.

An alternative explanation may derive from the deficits in social interaction, which are associated with dysfunction in similar fronto-striatal networks. Behavioural and physiological studies show that social behaviour expressed through facial mimicry is sensitive to reward value signals and directly correlates with autistic traits in general population (Sims et al., 2012). Neuroimaging studies of facial mimicry and emotional empathy, find that children with high-functioning autism fail to engage inferior frontal gyrus (pars opercularis) to the same extent as typically developing controls (Dapretto et al., 2006). While the language deficits in Asperger syndrome (AS) pertain mainly to the social use (Rinehart et al., 2002), it is possible that word production in this group reflects a peculiar strategy linked to social aspects of language. However, this interpretation is still speculative since we used a covert verbal fluency task during our fMRI study.

Nevertheless, our corroborated results suggest a more generic neurofunctional substrate to the language profiles observed in Asperger syndrome, originating in impairments of executive function and possible social interaction.

The pattern of effective connectivity observed in the AS group may also lead to set-switching impairments. Within- and cross-modal switching deficits are recognised in children with ASD relative to the typically developed matched controls (Reed and McCarthy, 2012) and, during visual attention, delayed fronto-cerebellar activations in the systems (Belmonte et al., 2009).

Overall, our effective connectivity results qualify a recently proposed overarching explanatory model of ASD, that of disrupted cortical connectivity (Kana et al., 2011) to incorporate the view that basal ganglia abnormalities have key roles within this model through the aberrant routing of the information among distributed cortical processing centres (Prat and Stocco, 2011). An important feature of our study is the integration of neuroimaging datasets and methodologies to provide fresh insight into neural substrates underlying the pathogenesis of ASD.

### 4.1 Limitations

Caution is required in generalisation of these results, since the precise relationship between grey matter textural parameters and the subjacent cytoarchitecture is yet to be systematically quantified. Progress in this is of course limited by availability of *postmortem* materials, however high field structural MRI may soon bridge this information gap. In addition, further neuroimaging studies will help define the functional consequences of grey-matter heterogeneity on brain activation across specific neurocognitive tasks.

While grey matter textural heterogeneity modulated the caudate–cortical connectivity in the PPI group-level analysis, the relationship of texture parameters with DCM parameters is less clear. However, PPI analysis with grey matter texture parameters guided the ROIs selection for the construction and testing the dynamic causal models. Likewise, a complementary way to explore the effect of grey-matter heterogeneity on connectivity is to examine the relationship between the textural-parameters and structural measures of white mater pathways (e.g. diffusion tensor imaging/fractional anisotropy).

We also acknowledge that it is premature to make generalised inferences about fronto-striatal ‘deficiency’ across multiple cognitive domains in AS without a more complex neuropsychological and neurophysiologic testing. A broader experimental approach specifically designed to show differences and similarities within and between ASDs and typically-developed individuals will help to better characterise the aberrant circuitry in AS.

Notwithstanding the above limitations, the corroborated findings from our present and previous studies on Asperger individuals (Beacher et al., 2012a, 2012b; Radulescu et al., 2013) represent a proof of concept in favour of informed multi-modal MRI approach in comprehensive characterisation of complex mental disorders. Specifically, the textural parameters expressing grey matter heterogeneity linked structural and functional modalities. Initially, these measures directed attention to structural differences in caudate volume in Asperger syndrome participants. Subsequently, these results served as basis for selecting the regions of interest for the effective connectivity analyses and helped highlighting disturbances in brain circuitry of language and visual attention in Asperger syndrome. These data converge on a disconnection account that emphasises the striatum as a critical structure in the brain basis of ASD.

In conclusion, our study combined textural analysis of grey matter images and fMRI effective connectivity techniques to show abnormalities in distributed fronto-striatal-parietal networks in Asperger syndrome, consistent with models of imbalanced top-down/bottom-up functional connections. Future studies may profit from focussing on these circuits. In particular, they may represent concrete targets for future diagnostic and therapeutical strategies in ASD.

The following are the supplementary data related to this article.

Supplementary material.

Supplementary Table 1 Participant specific MNI coordinates used for extracting the time series summaries (VOI) of the four regions used in the DCM specification.

Supplementary data to this article can be found online at http://dx.doi.org/10.1016/j.nicl.2013.05.010.

## Funding

This work was supported by the ‘Dr Mortimer and Theresa Sackler Foundation’ to ER (integrally), and HDC partially. This work was partly supported by the Wellcome Trust [grant number: 07433].

## Conflict of interest

BG, CC, RCDY provided texture analysis software (described in the manuscript) and have a commercial interest in the implementation of this textural analysis software in oncology related applications. BG, CC, RCDY did not participate to the data collection or analysis. ER, LM, NAH, MAG, FDCCB, HDC have no conflict of interest to declare in relation to the subject study.

## Figures and Tables

**Table 1:** Demographic and behavioural data.

	AS^a^ (N = 22; M/F^b^ = 11/11)	HV^c^ (N = 26; M/F^b^ = 12/14)	Total (N = 48; M/F^b^ = 23/25)
Age (mean ± SD)	32.45 ± 7.9 (Min = 21; max = 47)	29.46 ± 6.8 (Min = 19; max = 49)	30.83 ± 7.4 (Min = 19; max = 49)
NART^d^ (mean ± SD)	29.95 ± 7.9 (Min = 13; max = 40)	35.08 ± 6.3 (Min = 23; max = 44)	32.73 ± 7.5 (Min = 13; max = 44)
FLU^e^ performance (mean ± SD)	53.55 ± 16.2 (Min = 25; max = 89)	60.19 ± 18.5 (Min = 22; max = 96)	57.15 ± 17.64 (Min = 22; max = 96)
Handedness	L^f^ = 2; R^g^ = 19; N/A^h^ = 1	L^f^ = 3; R^g^ = 23	L^f^ = 5; R^g^ = 42; N/A^h^ = 1
Medication status	AD^i^ = 8 (M/F = 2/6)	AD^i^ = 2 (M/F = 1/1)	AD^i^ = 10 (M/F = 3/7)

Legend: AS^a^ = Asperger syndrome; M/F^b^: M = males, F = females; HV^c^ = healthy volunteers; NART^d^ = National Adult Reading Test; FLU^e^ = verbal fluency; L^f^ = left; R^g^ = right; N/A^h^ = not available; AD^i^ = antidepressants.

## References

[bb0005] Abutalebi J., Della Rosa P.A., Ding G., Weekes B., Costa A., Green D.W. (2013). Language proficiency modulates the engagement of cognitive control areas in multilinguals. Cortex.

[bb0010] Annese J. (2012). The importance of combining MRI and large-scale digital histology in neuroimaging studies of brain connectivity and disease. Frontiers in Neuroinformatics.

[bb0015] Barnea-Goraly N., Kwon H., Menon V., Eliez S., Lotspeich L., Reiss A.L. (2004). White matter structure in autism: preliminary evidence from diffusion tensor imaging. Biological Psychiatry.

[bb0020] Beacher F.D., Radulescu E., Minati L., Baron-Cohen S., Lombardo M.V., Lai M.C., Walker A., Howard D., Gray M.A., Harrison N.A., Critchley H.D. (2012). Sex differences and autism: brain function during verbal fluency and mental rotation. PloS One.

[bb0025] Beacher F.D., Minati L., Baron-Cohen S., Lombardo M.V., Lai M.C., Gray M.A., Harrison N.A., Critchley H.D. (2012). Autism attenuates sex differences in brain structure: a combined voxel-based morphometry and diffusion tensor imaging study. AJNR. American Journal of Neuroradiology.

[bb0030] Belmonte M.K., Gomot M., Baron-Cohen S. (2009). Visual attention in autism families: ‘unaffected’ sibs share atypical frontal activation. Journal of Child Psychology and Psychiatry.

[bb0035] Bitan T., Burman D.D., Lu D., Cone N.E., Gitelman D.R., Mesulam M.M., Booth J.R. (2006). Weaker top-down modulation from the left inferior frontal gyrus in children. NeuroImage.

[bb0040] Bitan T., Cheon J., Lu D., Burman D.D., Booth J.R. (2009). Developmental increase in top-down and bottom-up processing in a phonological task: an effective connectivity, fMRI study. Journal of Cognitive Neuroscience.

[bb0045] Broyd S.J., Demanuele C., Debener S., Helps S.K., James C.J., Sonuga-Barke E.J. (2009). Default-mode brain dysfunction in mental disorders: a systematic review. Neuroscience and Biobehavioral Reviews.

[bb0050] Casanova M., Trippe J. (2009). Radial cytoarchitecture and patterns of cortical connectivity in autism. Philosophical Transactions of the Royal Society of London.

[bb0055] Castellano G., Bonilha L., Li L.M., Cendes F. (2004). Texture analysis of medical images. Clinical Radiology.

[bb0060] Cavanna A.E., Trimble M.R. (2006). The precuneus: a review of its functional anatomy and behavioural correlates. Brain.

[bb0065] Chan S.H., Ryan L., Bever T.G. (2013). Role of the striatum in language: syntactic and conceptual sequencing. Brain and Language.

[bb0070] Chang C.W., Ho C.C., Chen J.H. (2012). ADHD classification by a texture analysis of anatomical brain MRI data. Frontiers in Systems Neuroscience.

[bb0075] Chumbley J., Worsley K., Flandin G., Friston K. (2010). Topological FDR for neuroimaging. NeuroImage.

[bb0080] Corbetta M. (1998). Frontoparietal cortical networks for directing attention and the eye to visual locations: identical, independent, or overlapping neural systems?. Proceedings of the National Academy of Sciences of the United States of America.

[bb0085] Dapretto M., Davies M.S., Pfeifer J.H., Scott A.A., Sigman M., Bookheimer S.Y., Iacoboni M. (2006). Understanding emotions in others: mirror neuron dysfunction in children with autism spectrum disorders. Nature Neuroscience.

[bb0260] de Oliveira M.S., Betting L.E., Mory S.B., Cendes F., Castellano G. (2013). Texture analysis of magnetic resonance images of patients with juvenile myoclonic epilepsy. Epilepsy & Behavior.

[bb0090] Dehaene S., Le Clec H.G., Poline J.B., Le Bihan D., Cohen L. (2002). The visual word form area: a prelexical representation of visual words in the fusiform gyrus. Neuroreport.

[bb0095] Eickhoff S.B., Walters N.B., Schleicher A., Kril J., Egan G.F., Zilles K., Watson J.D., Amunts K. (2005). High-resolution MRI reflects myeloarchitecture and cytoarchitecture of human cerebral cortex. Human Brain Mapping.

[bb0100] Eickhoff S.B., Laird A.R., Grefkes C., Wang L.E., Zilles K., Fox P.T. (2009). Coordinate-based activation likelihood estimation meta-analysis of neuroimaging data: a random-effects approach based on empirical estimates of spatial uncertainty. Human Brain Mapping.

[bb0105] Eickhoff S.B., Heim S., Zilles K., Amunts K. (2009). A systems perspective on the effective connectivity of overt speech production. Philosophical Transactions. Series A, Mathematical, Physical, and Engineering Sciences.

[bb0110] Elliott R. (2003). Executive functions and their disorders. British Medical Bulletin.

[bb0115] Friederici A.D. (2006). What's in control of language?. Nature Neuroscience.

[bb0125] Ganeshan B., Miles K.A., Young R.C., Chatwin C.R. (2008). Three-dimensional selective-scale texture analysis of computed tomography pulmonary angiograms. Investigative Radiology.

[bb0120] Ganeshan B., Miles K.A., Young R.C., Chatwin C.R., Gurling H.M., Critchley H.D. (2010). Three-dimensional textural analysis of brain images reveals distributed grey-matter abnormalities in schizophrenia. European Radiology.

[bb0130] Garbin G., Costa A., Sanjuan A., Forn C., Rodriguez-Pujadas A., Ventura N., Belloch V., Hernandez M., Avila C. (2011). Neural bases of language switching in high and early proficient bilinguals. Brain and Language.

[bb0135] Gazzaley A., Nobre A.C. (2012). Top-down modulation: bridging selective attention and working memory. Trends in Cognitive Sciences.

[bb0140] Geschwind D.H. (2008). Autism: many genes, common pathways?. Cell.

[bb0145] Gray M.A., Egan G.F., Ando A., Churchyard A., Chua P., Stout J.C., Georgiou-Karistianis N. (2013). Prefrontal activity in Huntington's disease reflects cognitive and neuropsychiatric disturbances: the IMAGE-HD study. Experimental Neurology.

[bb0150] Happe F., Frith U. (2006). The weak coherence account: detail-focused cognitive style in autism spectrum disorders. Journal of Autism and Developmental Disorders.

[bb0155] Hedden T., Gabrieli J.D. (2010). Shared and selective neural correlates of inhibition, facilitation, and shifting processes during executive control. NeuroImage.

[bb0160] Hilgetag C.C., Barbas H. (2006). Role of mechanical factors in the morphology of the primate cerebral cortex. PLoS Computational Biology.

[bb0165] Holli K.K., Harrison L., Dastidar P., Waljas M., Liimatainen S., Luukkaala T., Ohman J., Soimakallio S., Eskola H. (2010). Texture analysis of MR images of patients with mild traumatic brain injury. BMC Medical Imaging.

[bb0170] Jahfari S., Waldorp L., van den Wildenberg W.P., Scholte H.S., Ridderinkhof K.R., Forstmann B.U. (2011). Effective connectivity reveals important roles for both the hyperdirect (fronto-subthalamic) and the indirect (fronto-striatal–pallidal) fronto-basal ganglia pathways during response inhibition. Journal of Neuroscience.

[bb0175] Just M.A., Cherkassky V.L., Keller T.A., Minshew N.J. (2004). Cortical activation and synchronization during sentence comprehension in high-functioning autism: evidence of underconnectivity. Brain.

[bb0180] Kana R.K., Keller T.A., Cherkassky V.L., Minshew N.J., Just M.A. (2006). Sentence comprehension in autism: thinking in pictures with decreased functional connectivity. Brain.

[bb0185] Kana R.K., Keller T.A., Minshew N.J., Just M.A. (2007). Inhibitory control in high-functioning autism: decreased activation and underconnectivity in inhibition networks. Biological Psychiatry.

[bb0190] Kana R.K., Libero L.E., Moore M.S. (2011). Disrupted cortical connectivity theory as an explanatory model for autism spectrum disorders. Physics of Life Reviews.

[bb0195] Kassner A., Thornhill R.E. (2010). Texture analysis: a review of neurologic MR imaging applications. AJNR. American Journal of Neuroradiology.

[bb0200] Ketteler D., Kastrau F., Vohn R., Huber W. (2008). The subcortical role of language processing. High level linguistic features such as ambiguity-resolution and the human brain; an fMRI study. NeuroImage.

[bb0205] Langen M., Durston S., Staal W.G., Palmen S.J., van Engeland H. (2007). Caudate nucleus is enlarged in high-functioning medication-naive subjects with autism. Biological Psychiatry.

[bb0210] Langen M., Leemans A., Johnston P., Ecker C., Daly E., Murphy C.M., Dell'acqua F., Durston S., Murphy D.G. (2012). Fronto-striatal circuitry and inhibitory control in autism: findings from diffusion tensor imaging tractography. Cortex.

[bb0215] Lynch C.J., Uddin L.Q., Supekar K., Khouzam A., Phillips J., Menon V. (2013). Default mode network in childhood autism: posteromedial cortex heterogeneity and relationship with social deficits. Biological Psychiatry.

[bb0220] Mangin J.F., Jouvent E., Cachia A. (2010). In-vivo measurement of cortical morphology: means and meanings. Current Opinion in Neurology.

[bb0225] McAlonan G.M., Daly E., Kumari V., Critchley H.D., van Amelsvoort T., Suckling J., Simmons A., Sigmundsson T., Greenwood K., Russell A., Schmitz N., Happe F., Howlin P., Murphy D.G. (2002). Brain anatomy and sensorimotor gating in Asperger's syndrome. Brain.

[bb0230] McCandliss B.D., Cohen L., Dehaene S. (2003). The visual word form area: expertise for reading in the fusiform gyrus. Trends in Cognitive Sciences.

[bb0240] Minshew N.J., Keller T.A. (2010). The nature of brain dysfunction in autism: functional brain imaging studies. Current Opinion in Neurology.

[bb0235] Minshew N.J., Goldstein G., Siegel D.J. (1997). Neuropsychologic functioning in autism: profile of a complex information processing disorder. Journal of the International Neuropsychological Society.

[bb0245] Murty V.P., Sambataro F., Radulescu E., Altamura M., Iudicello J., Zoltick B., Weinberger D.R., Goldberg T.E., Mattay V.S. (2011). Selective updating of working memory content modulates meso-cortico-striatal activity. NeuroImage.

[bb0250] Nordahl C.W., Dierker D., Mostafavi I., Schumann C.M., Rivera S.M., Amaral D.G., Van Essen D.C. (2007). Cortical folding abnormalities in autism revealed by surface-based morphometry. Journal of Neuroscience.

[bb0255] Oh T.M., Tan K.L., Ng P., Berne Y.I., Graham S. (2011). The past tense debate: is phonological complexity the key to the puzzle?. NeuroImage.

[bb0265] Osechinskiy S., Kruggel F. (2009). Quantitative comparison of high-resolution MRI and myelin-stained histology of the human cerebral cortex. Conference Proceedings: … Annual International Conference of the IEEE Engineering in Medicine and Biology Society. IEEE Engineering in Medicine and Biology Society. Conference.

[bb0275] Penny W.D., Mattout J., Trujillo-Barreto N., Friston K., Ashburner J., Kiebel S., Nichols T., Penny W. (2006). Bayesian model selection and averaging. Statistical Parametric Mapping: the Analysis of Functional Brain Images.

[bb0270] Penny W.D., Stephan K.E., Daunizeau J., Rosa M.J., Friston K.J., Schofield T.M., Leff A.P. (2010). Comparing families of dynamic causal models. PLoS Computational Biology.

[bb0280] Pina-Camacho L., Villero S., Fraguas D., Boada L., Janssen J., Navas-Sanchez F.J., Mayoral M., Llorente C., Arango C., Parellada M. (2012). Autism spectrum disorder: does neuroimaging support the DSM-5 proposal for a symptom dyad? A systematic review of functional magnetic resonance imaging and diffusion tensor imaging studies. Journal of Autism and Developmental Disorders.

[bb0285] Prat C.S., Stocco A. (2011). Information routing in the basal ganglia: highways to abnormal connectivity in autism?: Comment on “disrupted cortical connectivity theory as an explanatory model for autism spectrum disorders” by Kana et al. Physics of Life Reviews.

[bb0290] Price C.J. (2010). The anatomy of language: a review of 100 fMRI studies published in 2009. Annals of the New York Academy of Sciences.

[bb0295] Price C.J., Devlin J.T. (2003). The myth of the visual word form area. NeuroImage.

[bb0300] Radulescu E., Ganeshan B., Minati L., Beacher F.D., Gray M.A., Chatwin C., Young R.C., Harrison N.A., Critchley H.D. (2013). Gray matter textural heterogeneity as a potential in-vivo biomarker of fine structural abnormalities in Asperger syndrome. The Pharmacogenomics Journal.

[bb0305] Reed P., McCarthy J. (2012). Cross-modal attention-switching is impaired in autism spectrum disorders. Journal of Autism and Developmental Disorders.

[bb0310] Rinehart N.J., Bradshaw J.L., Brereton A.V., Tonge B.J. (2002). A clinical and neurobehavioural review of high-functioning autism and Asperger's disorder. The Australian and New Zealand Journal of Psychiatry.

[bb0315] Robbins T.W. (2007). Shifting and stopping: fronto-striatal substrates, neurochemical modulation and clinical implications. Philosophical Transactions of the Royal Society of London.

[bb0320] Robinson J.L., Laird A.R., Glahn D.C., Blangero J., Sanghera M.K., Pessoa L., Fox P.M., Uecker A., Friehs G., Young K.A., Griffin J.L., Lovallo W.R., Fox P.T. (2012). The functional connectivity of the human caudate: an application of meta-analytic connectivity modeling with behavioral filtering. NeuroImage.

[bb0325] Rota G., Handjaras G., Sitaram R., Birbaumer N., Dogil G. (2010). Reorganization of functional and effective connectivity during real-time fMRI-BCI modulation of prosody processing. Brain and Language.

[bb0330] Sambataro F., Reed J.D., Murty V.P., Das S., Tan H.Y., Callicott J.H., Weinberger D.R., Mattay V.S. (2009). Catechol-O-methyltransferase valine(158)methionine polymorphism modulates brain networks underlying working memory across adulthood. Biological Psychiatry.

[bb0335] Sambataro F., Fazio L., Taurisano P., Gelao B., Porcelli A., Mancini M., Sinibaldi L., Ursini G., Masellis R., Caforio G. (2013). DRD2 genotype-based variation of default mode network activity and of its relationship with striatal DAT binding. Schizophrenia Bulletin.

[bb0340] Schipul S.E., Keller T.A., Just M.A. (2011). Inter-regional brain communication and its disturbance in autism. Frontiers in Systems Neuroscience.

[bb0345] Simard F., Monetta L., Nagano-Saito A., Monchi O. (2013). A new lexical card-sorting task for studying fronto-striatal contribution to processing language rules. Brain and Language.

[bb0350] Sims T.B., Van Reekum C.M., Johnstone T., Chakrabarti B. (2012). How reward modulates mimicry: EMG evidence of greater facial mimicry of more rewarding happy faces. Psychophysiology.

[bb0355] Stanfield A.C., McIntosh A.M., Spencer M.D., Philip R., Gaur S., Lawrie S.M. (2008). Towards a neuroanatomy of autism: a systematic review and meta-analysis of structural magnetic resonance imaging studies. European Psychiatry.

[bb0360] Stephan K.E., Penny W.D., Moran R.J., den Ouden H.E., Daunizeau J., Friston K.J. (2010). Ten simple rules for dynamic causal modeling. NeuroImage.

[bb0365] Takarae Y., Minshew N.J., Luna B., Sweeney J.A. (2007). Atypical involvement of frontostriatal systems during sensorimotor control in autism. Psychiatry Research.

[bb0370] Tan L.H., Chen L., Yip V., Chan A.H., Yang J., Gao J.H., Siok W.T. (2011). Activity levels in the left hemisphere caudate–fusiform circuit predict how well a second language will be learned. Proceedings of the National Academy of Sciences of the United States of America.

[bb0375] Theocharakis P., Glotsos D., Kalatzis I., Kostopoulos S., Georgiadis P., Sifaki K., Tsakouridou K., Malamas M., Delibasis G., Cavouras D., Nikiforidis G. (2009). Pattern recognition system for the discrimination of multiple sclerosis from cerebral microangiopathy lesions based on texture analysis of magnetic resonance images. Magnetic Resonance Imaging.

[bb0380] Turner K.C., Frost L., Linsenbardt D., McIlroy J.R., Muller R.A. (2006). Atypically diffuse functional connectivity between caudate nuclei and cerebral cortex in autism. Behavioral and Brain Functions.

[bb0385] van de Ven V., Esposito F., Christoffels I.K. (2009). Neural network of speech monitoring overlaps with overt speech production and comprehension networks: a sequential spatial and temporal ICA study. NeuroImage.

[bb0390] Van Essen D.C. (1997). A tension-based theory of morphogenesis and compact wiring in the central nervous system. Nature.

[bb0395] van Schouwenburg M.R., den Ouden H.E., Cools R. (2010). The human basal ganglia modulate frontal–posterior connectivity during attention shifting. Journal of Neuroscience.

[bb0400] Volkmar F.R., Klin A. (2000). Pervasive Developmental Disorders.

[bb0405] Wallace G.L., Dankner N., Kenworthy L., Giedd J.N., Martin A. (2010). Age-related temporal and parietal cortical thinning in autism spectrum disorders. Brain.

[bb0410] Whitney C., Weis S., Krings T., Huber W., Grossman M., Kircher T. (2009). Task-dependent modulations of prefrontal and hippocampal activity during intrinsic word production. Journal of Cognitive Neuroscience.

[bb0415] Zhang J., Yu C., Jiang G., Liu W., Tong L. (2012). 3D texture analysis on MRI images of Alzheimer's disease. Brain Imaging and Behavior.

