# Supplementary material for: Abnormalities in fronto-striatal connectivity within language networks relate to differences in grey-matter heterogeneity in Asperger syndrome
Source: Neuroimage Clin. 2013 May 27;2:716–26. doi: 10.1016/j.nicl.2013.05.010 (PMC3777793; doi:10.1016/j.nicl.2013.05.010)
Supplement: Supplementary file 1 — Supplementary material. [file mmc1.docx]

**Abnormalities in fronto-striatal connectivity within language networks relate to differences in grey-matter heterogeneity in Asperger Syndrome**

Eugenia Radulescu, MD PhD^1,2^, Ludovico Minati, PhD^3,4^, Balaji Ganeshan, PhD^1,5^, Neil A Harrison, MBBS^1,2,6^ PhD, Marcus A Gray, PhD^2,7^, Felix DCC Beacher, PhD^1^, Chris Chatwin, PhD^8^, Rupert CD Young, PhD^8^_,_ and Hugo D Critchley, MBChB, DPhil^1,2,6^

1. **Supplementary Method**

*1.1 Psycho-Physiological Interaction (PPI)*

PPIs represent ~~simple~~ models of ~~effective~~ **functional** connectivity. They quantify the change in contribution of one brain area to another, when manipulating the experimental context (Friston et al., 1997, Gitelman et al., 2003). Essentially, whole-brain connectivity is calculated between the time-series of a seed/source region and the time-series of all the other voxels activated during an experimental condition. For performing the PPI, we used a standard protocol (http://www.fil.ion.ucl.ac.uk/spm/): 1. First level voxel-wise analysis for effects and confounds of verbal fluency task performance (the individual GLMs described in Beacher et al., 2012a); 2. Extracting the BOLD signal from the region of interest/ source (in our case the caudate nuclei); 3. Calculation of the interaction term (deconvolved source signal x experimental manipulation); 4. Performing a second GLM analysis with experimental vectors, reconvolved interaction term and the source region’s extracted BOLD times series as regressors.

*1.1.1 Signal extraction for PPI*

We used the time series of the left and right caudate extracted in each participant from the positive T contrast of the ‘letter’ condition inclusively masked with an image provided by the Automated Anatomical Labelling (AAL) library implemented in the Wake Forest University Pickatlas (WFU) (<http://www.fmri.wfubmc.edu>). The mask image comprised the head and body of caudate, separately for right and left side.

Within the mask, we selected the caudate cluster and extracted the time-series summary (first eigenvariate- VOI) from an 8 mm sphere centred at the cluster’s peak coordinates, after adjusting for effects of interest. We used for VOI extraction a lenient statistical threshold (0.05≤ p< 0.07 uncorrected) due to the substantial intra-subject variability in caudate recruitment during word generation. This variability is probably related to the more effortful nature of phonemic fluency process that is not a current strategy in language production and can be influenced by various factors (i.e. bilingualism, Luo et al., 2010; disease, Thames et al., 2012, etc.).

*1.1.2 Creating the PPI regressors*

The interactions at brain level are produced by the neural signal in the related regions. However, in the usual GLM analysis, the neural signal is convolved with a hemodynamic response function. Consequently, a deconvolution step is necessary to indirectly derive the neural signal and calculate the interaction term (Gitelman et al., 2003). To perform this step and create the experimental vectors and the interaction product we used the SPM8 PPI software.

The time series of the caudate neuronal signals for word generation (‘letter’) condition and visual attention (‘control’) condition were created, resulting in two PPI regressors (two vectors), one for each experimental condition.

In addition, one PPI interaction term was computed for each experimental condition as the product between the source region’s extracted signal and the corresponding experimental vector. The PPI interaction term represents the manner by which an area’s response to the experimental conditions is modulated by the input from another region.

*1.2.3 GLM analysis with PPI regressors*

The interactions terms were subsequently introduced in a general linear model (regression), together with the original VOI time series, and the main effects of the two experimental conditions (experimental vectors), after re-convolution with the HRF. The six motion parameters were also introduced as covariates of non-interest.

After model estimation, the SPMs revealed the areas connected with the VOIs due to the context manipulations. Individual contrasts between PPI terms of ‘letter’ versus ‘control’ were computed and used in second-level group analyses.

*1.2 Dynamic Causal Modelling (DCM)*

*1.2.1 Time series extraction*

For each participant, 6 mm spheres (VOIs) were specified as the local maxima within the L-IFG, R-IFG, caudate (‘letter’ condition, F contrast) and from within the precuneus (‘control’ condition, F contrast). We opted for extracting the summary of the time series (first principal eigenvariate) from the F contrast because we assumed variability across subjects and were interested in finding any change in BOLD signal irrespective of directionality (positive or negative). Likewise, the selection was guided by the group analysis results, such that the VOI coordinates within the WFU defined ROIs, did not differ from the group coordinates by > 6 mm in any direction, as previously recommended (i.e. Leff et al 2008). Consequently, 5/48 participants who did not meet the above criterion were discarded from the subsequent DCM construction (controls: one male and one female; AS: two males and one female). For a complete list of VOI coordinates see **Supplementary table 2**.

*1.2.2 Defining the inputs (GLM- matrix design for DCM)*

The design matrix for DCM comprised the same conditions and regressors as the initial GLM with the exception of one additional regressor-‘All stimuli’- including the onsets for ‘letter’ and ‘control’ conditions collapsed. The purpose of this regressor was the specification of driving inputs for the R-IFG/ Ins and was motivated by the strong BOLD response in this area during both conditions, ‘letter’ and ‘control’. The movement parameters were introduced as covariates of non-interest. After defining the inputs based on the GLM design matrix and extracting the time series, we built bilinear deterministic DCMs (Friston et al 2003) implemented in DCM10. In bilinear DCM, three types of parameters are estimated: a) driving inputs, representing the direct influence of stimuli on the regional activity; b) intrinsic connections between the selected regions, independent of experimental manipulation; c) effects of experimental conditions on regional activity or on connections between regions (modulatory effects).

*1.2.3 Defining the Dynamic Causal Models (DCMs) featuring the backward (top-down) and forward (bottom-up) connections*

In each of the first four DCMs, we modelled the driving inputs represented the by the effect of ‘All stimuli’ on the R-IFG/ Ins, the intrinsic connections and the effects of ‘Letter’ condition on the L- IFG, respectively of the ‘Control’ condition on the precuneus . As mentioned in the body text, the location of driving inputs was informed by the functional GLM, PPI and previous studies (Price 2010; Corbetta 1998).

In the first model, we specified all the possible bidirectional connections between the ROIs. Thereafter, we eliminated inter-regional connections, firstly the direct bidirectional connections between frontal regions and precuneus, while keeping the caudate as a relay between frontal and parietal (precuneus) (model 2, **figure 2** in the body text). In the third and fourth models we represented unidirectional ‘top-down’ connections, from frontal to precuneus, respectively ‘bottom-up’, from the precuneus to frontal, also maintaining the caudate as relay between the frontal regions and the precuneus (**figure 2** in the body text).

*1.2.4 Defining the Dynamic Causal Models (DCMs) featuring the modulatory effects of the experimental ‘letter’ condition.*

Six models based on two main architectures were created: three models testing the effect of ‘letter’ (the condition of interest) on each top-down connection (L-IFG to caudate; R-IFG to caudate; caudate to precuneus), respectively three models testing the effect of ‘letter’ on each bottom-up connection (precuneus to caudate; caudate to L-IFG; caudate to R-IFG) (**figure 2** in the body text).

Each of the ten models was estimated at individual level by a fixed-effect analysis under a Bayesian Model Selection (BMS) procedure (Stephan et al 2010).

1. **Supplementary bibliography:**

M. Corbetta, Frontoparietal cortical networks for directing attention and the eye to visual locations: identical, independent, or overlapping neural systems?, Proc Natl Acad Sci U S A 95 (1998) 831-838.

K.J. Friston, C. Buechel, G.R. Fink, J. Morris, E. Rolls, R.J. Dolan, Psychophysiological and modulatory interactions in neuroimaging, NeuroImage 6 (1997) 218-229.

K.J. Friston, L. Harrison, W. Penny, Dynamic causal modelling, NeuroImage 19 (2003) 1273-1302.

D.R. Gitelman, W.D. Penny, J. Ashburner, K.J. Friston, Modeling regional and psychophysiologic interactions in fMRI: the importance of hemodynamic deconvolution, NeuroImage 19 (2003) 200-207.

A.P. Leff, T.M. Schofield, K.E. Stephan, J.T. Crinion, K.J. Friston, C.J. Price, The cortical dynamics of intelligible speech, J Neurosci. 28 (2008) 13209-13215.

L. Luo, G. Luk, E. Bialystok, Effect of language proficiency and executive control on verbal fluency performance in bilinguals, Cognition 114 (2010) 29-41.

C.J. Price, The anatomy of language: a review of 100 fMRI studies published in 2009, Ann N Y Acad Sci. 1191 (2010) 62-88.

K.E. Stephan, W.D. Penny, R.J. Moran, H.E. den Ouden, J. Daunizeau, K.J. Friston, Ten simple rules for dynamic causal modeling, NeuroImage 49 (2010) 3099-3109.

A.D. Thames, J.M. Foley, M.J. Wright, S.E. Panos, M. Ettenhofer, A. Ramezani, V. Streiff, S. El-Saden, S. Goodwin, S.Y. Bookheimer, C.H. Hinkin, Basal ganglia structures differentially contribute to verbal fluency: evidence from Human Immunodeficiency Virus (HIV)-infected adults, Neuropsychologia 50 (2012) 390-395.
