# Supplementary material for: Abnormalities in fronto-striatal connectivity within language networks relate to differences in grey-matter heterogeneity in Asperger syndrome
Source: Neuroimage Clin. 2013 May 27;2:716–26. doi: 10.1016/j.nicl.2013.05.010 (PMC3777793; doi:10.1016/j.nicl.2013.05.010)
Supplement: Supplementary Table 1 — Participant specific MNI coordinates used for extracting the time series summaries (VOI) of the four regions used in the DCM specification. [file mmc2.docx]

**Supplementary Table 1:** Participant specific MNI coordinates used for extracting the time series summaries (VOI) of the four regions used in the DCM specification.

| **Participants** | **L- Inferior Frontal Gyrus* (x y z coordinates)** | **R- Inferior Frontal Gyrus/ Insula** (x y z coordinates)** | **Caudate (x y z coordinates)** | **Precuneus (x y z coordinates)** |
| --- | --- | --- | --- | --- |
| 1. Control males | -46 4 22 | 36 4 34 | 16 16 0 | 2 -74 28 |
| 1. Control males | -34 6 32 | 38 6 28 | 4 14 6 | -20 -64 36 |
| 1. Control males | -34 24 20 | 56 18 36 | -6 16 -6 | 32 -86 34 |
| 1. Control males | -42 20 12 | 62 14 16 | -4 12 6 | 0 -54 54 |
| 1. Control males | -48 6 26 | 38 10 30 | 10 16 -2 | 14 -66 26 |
| 1. Control males | -34 24 32 | 38 14 32 | 18 18 6 | 6 -72 16 |
| 1. Control males | -34 20 16 | 52 14 36 | 6 4 2 | -26 -64 34 |
| 1. Control males | -44 8 30 | 38 14 32 | 8 12 6 | 2 -72 26 |
| 1. Control males | -42 0 30 | 50 18 38 | -2 14 2 | 0 -74 26 |
| 1. Control males | -48 2 24 | 42 24 30 | 8 6 2 | 4 -74 20 |
| 1. Control males | -40 22 20 | 44 10 36 | 12 16 -6 | 4 -74 42 |
| 1. Control females | -50 -4 32 | 46 18 26 | 10 16 -6 | 28 -78 16 |
| 1. Control females | -50 -4 32 | 42 8 28 | -4 2 0 | 10 -84 44 |
| 1. Control females | -54 -4 28 | 46 6 0 | 18 16 6 | 2 -78 24 |
| 1. Control females | -54 -4 24 | 54 24 32 | 14 16 6 | 2 -78 24 |
| 1. Control females | -54 2 32 | 44 12 30 | 6 4 4 | 0 -76 20 |
| 1. Control females | -42 2 32 | 44 8 28 | -2 12 -2 | 2 -78 26 |
| 1. Control females | -42 6 12 | 36 8 10 | 16 20 2 | -18 -74 44 |
| 1. Control females | -38 2 32 | 40 4 26 | 14 20 2 | 14 -60 22 |
| 1. Control females | -48 12 32 | 38 12 30 | 8 12 -6 | -2 -74 48 |
| 1. Control females | -52 6 32 | 40 6 34 | -12 16 6 | 10 -78 40 |
| 1. Control females | -38 6 22 | 38 16 30 | 12 6 2 | -10 -74 20 |
| 1. Control females | -38 10 18 | 56 12 20 | 16 14 2 | -14 -62 38 |
| 1. Control females | -34 2 32 | 38 14 26 | 10 22 0 | -4 -80 38 |
| 1. AS males | -44 24 14 | 26 20 -16 | 8 20 -2 | 16 -70 20 |
| 1. AS males | -46 16 26 | 46 10 38 | -2 10 6 | 6 -72 42 |
| 1. AS males | -36 24 28 | 40 18 32 | -2 6 -2 | -22 -78 34 |
| 1. AS males | -54 6 32 | 38 6 30 | 12 8 6 | 0 -76 20 |
| 1. AS males | -52 12 26 | 42 4 26 | -10 20 0 | -30 -72 38 |
| 1. AS males | -54 24 24 | 40 6 32 | -6 20 -6 | 36 -74 34 |
| 1. AS males | -42 10 32 | 60 12 24 | 14 14 2 | -16 -70 28 |
| 1. AS males | -38 8 24 | 46 20 30 | 16 14 6 | 8 -74 20 1 |
| 1. AS males | -38 8 28 | 38 8 34 | 16 24 2 | 2 -76 34 |
| 1. AS females | -50 -4 22 | 52 20 26 | 4 12 6 | -26 -78 40 |
| 1. AS females | -52 24 20 | 42 12 30 | 6 8 6 | -8 -84 44 |
| 1. AS females | -42 0 32 | 56 20 12 | 14 16 2 | 10 -80 46 |
| 1. AS females | -38 10 32 | 34 14 -16 | 4 8 -2 | 2 -78 40 |
| 1. AS females | -48 8 32 | 54 22 32 | 10 8 0 | -26 -58 54 |
| 1. AS females | -50 6 30 | 46 14 32 | 6 4 6 | -10 -74 40 |
| 1. AS females | -38 24 32 | 46 12 -12 | 14 14 6 | -20 -52 54 |
| 1. AS females | -50 8 26 | 56 12 26 | -6 2 6 | -26 -76 40 |
| 1. AS females | -34 22 22 | 46 4 -8 | -10 16 -2 | 24 -60 42 |
| 1. AS females | -38 24 32 | 44 8 28 | 12 12 -4 | 20 -74 52 |

***L- Inferior Frontal Gyrus** defined according to WFU Pickatlas labels (<http://fmri.wfubmc.edu/software/PickAtlas>) and group activation during the ‘Letter’ condition, encompasses the posterior part of pars opercularis and pars triangularis at intersection with left insula and premotor cortex.

****R- Inferior Frontal Gyrus** defined according to WFU Pickatlas labels and group activation during the ‘Letter’ condition, includes the right pars opercularis and right insula.

Details about Caudate and Precuneus in the body text.

*Abbreviations:* MNI= ‘Montreal Neurological Institute’; VOI= ‘volume of interest’ for extracting the first eigenvariate of the time series; DCM= ‘dynamic causal modelling’; AS= Asperger syndrome; L=left; R=right.
